# Transcriptomic analysis of ‘Suli’ pear (*Pyrus pyrifolia* white pear group) buds during the dormancy by RNA-Seq

**DOI:** 10.1186/1471-2164-13-700

**Published:** 2012-12-12

**Authors:** Guoqin Liu, Wanshun Li, Penghua Zheng, Tong Xu, Lijuan Chen, Dongfeng Liu, Sayed Hussain, Yuanwen Teng

**Affiliations:** 1 Department of Horticulture, The State Agricultural Ministry’s Key Laboratory of Horticultural Plant Growth, Development & Quality Improvement, Zhejiang University, Hangzhou, 310058,, Zhejiang Province, China; 2 College of Agriculture, Guizhou University, Guiyang, 550025,, Guizhou Province, China; 3 BGI-tech, Shenzhen, 518083,, Guangdong Province, China

**Keywords:** ‘Suli’ pear (*Pyrus pyrifolia* white pear group), Transcriptome, Bud dormancy, RNA-Seq

## Abstract

**Background:**

Bud dormancy is a critical developmental process that allows perennial plants to survive unfavorable environmental conditions. Pear is one of the most important deciduous fruit trees in the world, but the mechanisms regulating bud dormancy in this species are unknown. Because genomic information for pear is currently unavailable, transcriptome and digital gene expression data for this species would be valuable resources to better understand the molecular and biological mechanisms regulating its bud dormancy.

**Results:**

We performed *de novo* transcriptome assembly and digital gene expression (DGE) profiling analyses of ‘Suli’ pear (*Pyrus pyrifolia* white pear group) using the Illumina RNA-seq system. RNA-Seq generated approximately 100 M high-quality reads that were assembled into 69,393 unigenes (mean length = 853 bp), including 14,531 clusters and 34,194 singletons. A total of 51,448 (74.1%) unigenes were annotated using public protein databases with a cut-off E-value above 10^-5^. We mainly compared gene expression levels at four time-points during bud dormancy. Between Nov. 15 and Dec. 15, Dec. 15 and Jan. 15, and Jan. 15 and Feb. 15, 1,978, 1,024, and 3,468 genes were differentially expressed, respectively. Hierarchical clustering analysis arranged 190 significantly differentially-expressed genes into seven groups. Seven genes were randomly selected to confirm their expression levels using quantitative real-time PCR.

**Conclusions:**

The new transcriptomes offer comprehensive sequence and DGE profiling data for a dynamic view of transcriptomic variation during bud dormancy in pear. These data provided a basis for future studies of metabolism during bud dormancy in non-model but economically-important perennial species.

## Background

Dormancy is a complex phase of plant development that is necessary for survival under unfavorable environmental conditions. According to Lang [1], dormancy is a temporary suspension of visible growth of any plant structures containing meristems and can be divided into five well-defined phases: paradormancy, endodormancy, ecodormancy, and the two transitional phases between para- and endodormancy and endo- and ecodormancy. Dormancy transitions are regulated by short photoperiods and/or chilling temperatures. In pear low temperatures have been proven to control dormancy [2]. Several excellent studies have investigated the physiological and molecular mechanisms of bud-dormancy transitions in perennial woody and herbaceous plants, including leafy spurge (*Euphorbia esula* Linn.) [3-5], poplar (*Populus* spp.) [6,7], peach (*Prunus persica* [L.] Batsch) [8-12], apple (*Malus × domestica* Borkh) [13,14], Japanese apricot (*Prunus mume* Siebold Zucc.) [15,16], chestnut (*Castanea sativa* Mill.) [17-19], grape (*Vitis vinifera* linn.) [20,21], raspberry (*Rubus idaeus* Linn.) [22], kiwifruit (*Actinidia* spp.) [23,24], and blackcurrant (*Ribes nigrum* Linn.) [25]. These results suggested that bud dormancy involves many biochemical pathways related to photoperiod, temperature, circadian clocks, water, energy, reactive oxygen species, and hormones. Several genes involved in dormancy transition have been identified, providing useful references for studying perennial plant dormancy. However, many unresolved questions remain about how many pathways are involved, how they interact, and significant differences in dormancy transition and regulation among species, genotypes, tissues, and environments.

Pears (*Pyrus* spp.) are among the world's most important perennial deciduous fruit trees and have a key feature of transition from growth to dormancy during their annual growth cycles. Studies of pear dormancy have focused mainly on the physiological level, including respiration [26], carbohydrate [27,28] and protein metabolism [29], and chilling requirements [30]. To date, few studies of pear dormancy at the molecular level have been conducted. Ubi et al. (2010) isolated two dormancy-associated MADS-box (DAM) genes and studied their expression patterns during the seasonal endodormancy transition phases in Japanese pear (*Pyrus pyrifolia*) [31]. Although these data highlighted the potential of molecular research to understand dormancy in this crop, they were insufficient to elucidate the molecular regulation mechanism. Furthermore, with global warming, many fruit trees, including pears, growing in warm areas have suffered from inadequate winter chill and showed advanced springtime and delayed autumnal phenologies, uneven foliation and blossoming, and long blooming periods, which are unfavorable for sustainable pear production [32-37]. Therefore, understanding the molecular mechanisms of pear dormancy transitions will greatly assist programs to breed cultivars with lower chilling requirements and to develop agronomic measures to cope with insufficient winter chill.

Traditionally, researchers have studied target nucleotide sequences by cloning, sequencing and comparing with known sequences, annotating their functions, then verifying their functions using tools such as RNAi, microarrays, and genetic transformation. These methods are very useful, but characterizing a large number of genes in a single experiment is difficult, especially with respect to specific genes. In recent years, RNA-sequencing (RNA-Seq) technology based on pyro- sequencing has become the most popular and powerful tool for species that lack reference genome information. RNA-Seq is less costly, more efficient, and allows faster gene discovery and more sensitive and accurate profiles of the transcriptome than microarray analysis or other techniques [38-45]. To better understand the molecular mechanisms of bud dormancy transition in pear trees, we used RNA-Seq technology to identify and characterize the expression of a large number of genes, especially those differentially expressed during dormancy progression.

The aim of the present study was to gain an understanding of molecular mechanisms during pear bud dormancy and establish a sound foundation for future molecular studies. We sequenced cDNA libraries from lateral flower buds of ‘Suli’ pear (*Pyrus pyrifolia* white pear group) from endo to ecodormancy stages using Illumina deep-sequencing technology. Approximately 100 M high-quality reads were obtained and assembled into 69,393 unigenes. Furthermore, four digital gene expression (DGE) libraries were constructed to compare gene expression patterns in different dormancy states using an upgraded digital gene expression system. The assembled and annotated transcriptome sequences and gene expression profiles will provide valuable resources for the identification of pear genes involved in bud dormancy.

## Results

### Dormancy status of lateral flower buds in pears

**Figure 1:**
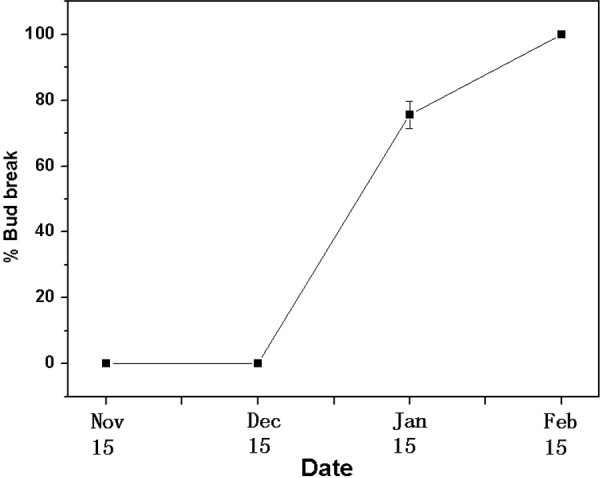
**Bud break percentage of 'Suli' pear after 21 days of forcing conditions.** Dormant shoots of field-grown ‘Suli’ pear trees were collected on Nov. 15, Dec. 15, Jan. 15, and Feb. 15, 2010–2011, and kept in water in a phytotron at day/night 25 ± 1/18 ± 1°C, with a 12-h photoperiod of white light (320 μ photon mol m^-2^ s^-1^) and 75% humidity. Percent bud break in 12 shoots per sampling period was assessed after 21 d.

To identify differentially expressed genes (DEGs) during dormancy, the dormancy status of lateral flower buds in pears must be defined. Dormancy status on four collection dates was estimated using excised shoots. No bud break was observed on shoots sampled on Nov. 15 or Dec. 15, but more than 50% of the buds had broken on Jan. 15 and Feb. 15 (Figure 1). Lateral flower buds were determined to be in the endodormant phase on Nov. 15 and Dec. 15 and in the ecodormant phase on Jan. 15 and Feb. 15.

### Illumina sequencing and *de novo* assembly

To obtain an overview of the pear bud transcriptome during dormancy, a cDNA library was generated from RNA isolated from buds pooled from Nov. 2010 to Feb. 2011, then paired-end sequenced using the Illumina platform. After cleaning and quality checks, approximately 100 M high-quality reads were assembled into 197,357 contigs (Table 1). The mean contig size was 272 bp. Using paired-end joining and gap-filling, these contigs were further assembled into 69,393 unique sequences with a mean size of 853 bp, including 14,531 clusters and 34,194 singletons. The size distributions of these contigs and unigenes are shown in Additional file 1. To evaluate the quality of sequencing data, we randomly selected 8 unigenes and designed 8 pairs of primers (Additional file 2) for RT-PCR amplification. Each primer pair resulted in a band of the expected size; the identity of all PCR products was confirmed by Sanger sequencing.

**Table 1:** Summary of the 'Suli' pear transcriptome during bud dormancy based on the RNA-Seq data

Total number of reads	39,757,914
Total base pairs (bp)	3,578,212,260
Average read length (bp)	90
Total number of contigs	197,357
Mean length of contigs (bp)	272
Total number of unigenes	69,393
Mean length of unigenes (bp)	853
Distinct clusters	14,531
Distinct sigletons	34,194
Sequences with E-value < 10^-5^	51,448

### Annotation of predicted proteins

**Figure 2:**
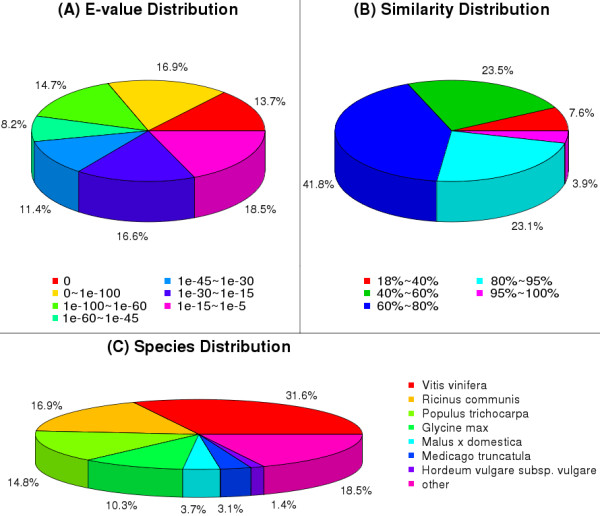
**Characteristics of homology search of pear unigenes against the nr database.** (**A**) E-value distribution of the top BLAST hits for each unique sequence. (**B**) Similarity distribution of the top BLAST hits for each unique sequence. (**C**) Species distribution of the top BLAST hits for all homologous sequences.

Approximately 51,448 unique sequences were annotated based on BLASTx (cut-off E-value 10^-5^) searches of four public databases: NCBI non-redundant (nr) database, Swiss-Prot protein database, Kyoto Encyclopedia of Genes and Genomes (KEGG) database, and Gene Ontology (GO) database (Additional file 3). Among them, 47,993 unique sequences could be annotated with reference to the nr database. Based on the nr annotations, 45.3% of the annotated sequences had very strong homology (E-value < 10^-60^), and 19.6% showed strong homology (10^-60^ < E-value < 10^-30^), and an additional 35.1% showed homology (10^-30^ < E-value < 10^-5^), to available plant sequences (Figure 2A). The similarity distribution was comparable, with 27.0% of the sequences having similarities higher than 80%, while 73.0% of the hits had similarities of 18–80% (Figure 2B). With respect to species, 31.6% of the unique sequences had top matches to sequences from *Vitis vinifera*, with additional hits to *Ricinus communis* (16.9%), *Populus trichocarpa* (14.8%), *Glycine max* (10.3%), and *Malus × domestica* (3.7%) (Figure 2C).

### Functional classification

**Figure 3:**
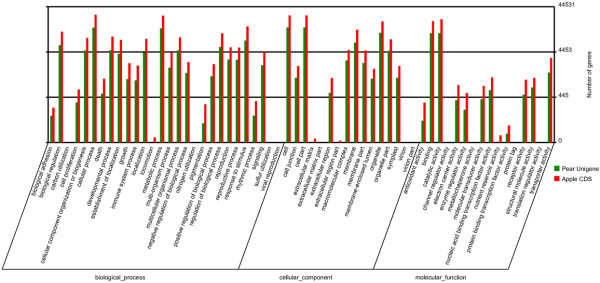
**Histogram of gene ontology classifications for pear and apple.** The unigenes corresponded to three main categories: biological process, cellular component, and molecular function. The right-hand y-axis indicates the number of annotated unigenes from pear and apple in a category.

We used GO and KEGG assignments to classify the functions of the predicted pear unigenes. Approximately 48,725 sequences could be annotated using GO, and 36,717 unigenes could be categorized into three main categories: biological process, cellular component, and molecular function. To our knowledge, the apple (*Malus × domestica*) genome has been completed. Among the organisms with completely-sequenced genomes, apple is most closely related to pear. Therefore, the distribution of GO annotations in the pear unigene data was compared with that of the apple genome (63,517 full length sequences) (ftp://ftp.jgi-psf.org/pub/JGI_data/phytozome/v8.0/Mdomestica/annotation/Mdomestica_196_cds.fa.gz) using Blast2GO. The sequences could be categorized into 60 GO functional groups (Figure 3). The percentages of annotated apple genes assigned to each group generally mirrored those of pear unigenes, reflecting the similar functionalities of their genomes and further highlighting that a large diversity of pear unigenes was represented by these sequences. Through sequence comparison, we observed that, although both species were highly similar, significant differences also existed between them.

There were 29,500 unigenes (approximately 42.5%) that mapped into 128 KEGG pathways (Additional file 4). The maps with highest unigene representation were metabolic pathways (KO 01100; 7,043 unigenes, 25.1%), followed by biosynthesis of secondary metabolites (KO 01110; 3,349 unigenes, 11.4%), plant-pathogen interactions (KO 04626; 1,531 unigenes, 5.2%), and plant hormone signal transduction (KO 04075; 1,357 unigenes, 4.6%).

### DGE library sequencing and mapping sequences to the reference transcriptome database

**Figure 4:**
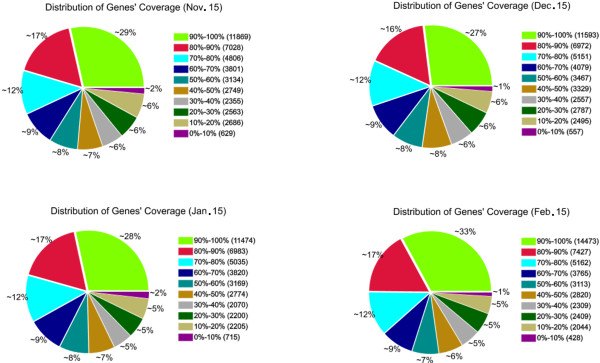
**Percent coverage representing the percentage of unigenes mapped in our transcriptome data that were expressed at four times during dormancy.** Gene coverage is the percentage of a gene covered by reads. This value is the ratio of the number of bases in a gene covered by unique mapping reads to the total bases in that gene. The distribution of unique reads over different read abundance categories show similar patterns for all four RNA-Seq libraries.

Using the RNA-seq technique, we analyzed changes in gene expression at four times during pear bud dormancy. Four DGE libraries (from buds sampled on Nov. 15, Dec. 15, Jan. 15, and Feb. 15) were sequenced to generate approximately 13–15 million clean reads per library after filtering the raw reads. The total number of mapped reads in each library ranged from 11.0–12.3 million, and the percentage of these reads ranged from 78.8–80.9%. Among them, the number of unique match reads ranged from 7.0–7.9 million (Table 2). To confirm whether the number of detected genes increased proportionally to sequencing effort, sequence saturation analysis was performed. A trend of saturation where the number of detected genes almost ceases to increase when the number of reads reaches 5 million (Additional file 5). We evaluated the randomness of the DGE data by analyzing the distribution of reads matching the reference genes [38], because nonrandom biases for specific gene regions can directly affect subsequent bioinformatics analysis. The randomness of the data was good, with reads evenly distributed throughout the transcriptome (Additional file 6). For mRNA expression, heterogeneity and redundancy are two significant characteristics. While the majority of mRNA is expressed at low levels, a small proportion of mRNA is highly expressed. Therefore, the distribution of unique reads was used to evaluate the normality of our RNA-Seq data. As shown in Figure 4, the distribution of unique reads over different reads abundance categories showed similar patterns for all four RNA-Seq libraries. The similarity distribution had a comparable pattern with more than 43% of the sequences having a similarity of 80%, while approximately 50% of the hits had a similar range (Figure 4).

**Table 2:** Summary of read numbers based on the RNA-Seq data from 'Suli' pear during bud dormancy

**Summary**	**Nov. 15**	**Dec. 15**	**Jan. 15**	**Feb. 15**
Total Clean Reads	15,156,144	15,250,358	14,364,828	13,920,719
Total Mapped Reads	12,267,906	12,336,227	11,604,366	10,973,976
Total Mapped Reads / Total Clean Reads	80.94%	80.89%	80.78%	78.83%
Perfect Match Reads	9,305,310	9,327,977	8,832,506	8,222,365
Perfect Match Reads / Total Clean Reads	61.40%	61.17%	61.49%	59.07%
≤2 bp Mismatch Reads	2,962,596	3,008,250	2,771,860	2,751,611
≤2 bp Mismatch Reads / Total Clean Reads	19.55%	19.73%	19.30%	19.77%
Unique Match Reads	7,902,930	7,977,788	7,457,487	7,042,745
Unique Match Reads / Total Clean Reads	52.14%	52.31%	51.91%	50.59%
Multi-Position Match Reads	4,364,976	4,358,439	4,146,879	3,931,231
Multi-Position Match Reads / Total Clean Reads	28.80%	28.58%	28.87%	28.24%
Total-Unmapped Reads	2,888,238	2,914,131	2,760,462	2,946,743
Total-Unmapped Reads / Total Clean Reads	19.06%	19.11%	19.22%	21.17%

### Changes in gene expression profiles among dormancy stages

**Figure 5:**
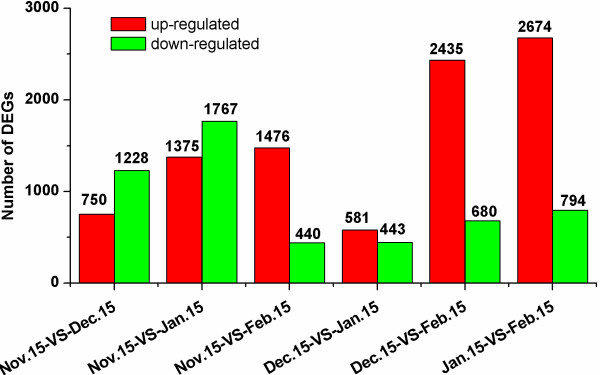
**Changes in gene expression profiles during different dormancy stages.** The numbers of up- and down-regulated genes in comparisons of the Nov. 15-VS-Dec. 15, Nov. 15-VS-Jan. 15, Nov. 15-VS-Feb. 15, Dec. 15-VS-Jan. 15, Dec. 15-VS-Feb. 15, and Jan. 15-VS-Feb. 15 libraries were summarized. DEGs: Differentially-expressed genes.

Differences in gene expression at four times during pear bud dormancy were examined, and DEGs were identified by pairwise comparisons of the four libraries, i.e. Nov. 15-VS-Dec. 15, Nov. 15-VS-Jan. 15, Nov. 15-VS-Feb. 15, Dec. 15-VS-Jan. 15, Dec. 15-VS-Feb. 15, and Jan. 15-VS-Feb. 15 (Figure 5 & Additional file 7, Additional file 8, Additional file 9, Additional file 10, Additional file 11, Additional file 12, respectively). Although lateral flower buds sampled on Nov. 15 and Dec. 15 were both in the endodormant stage, 1,978 genes were significantly differentially expressed between these libraries. Of these, 1,228 were down-regulated and 750 were up-regulated. Between the Dec. 15 and Jan. 15 libraries, there were 1,024 DEGs, with 443 down-regulated and 581 up-regulated. A total of 3,468 DEGs were detected between the Jan. 15 and Feb. 15 libraries, with 794 down-regulated and 2,674 up-regulated, although lateral flower buds sampled on both dates were in the endodormant-released stage. The greatest number of differentially-expressed genes occurred in the Jan. 15-VS-Feb. 15 comparison, followed by Nov. 15-VS-Jan. 15, Dec. 15-VS-Feb. 15, Nov. 15-VS-Feb. 15, Nov. 15-VS-Dec. 15, and Dec. 15-VS-Jan. 15.

We analyzed the functions of the most differentially-expressed genes with the expression fold (log_2_Ratio ≥ 2) and false discovery rate (FDR ≤ 10^-5^) as the threshold in the Nov. 15-VS-Dec. 15, Dec. 15-VS-Jan. 15, and Jan. 15-VS-Feb. 15 comparisons (Additional file 13) based on NCBI annotations. In the Nov. 15-VS-Dec. 15 comparison, only two of the 20 most up-regulated genes (stachyose synthase precursor and phytosulfokines precursor) had defined functions in the NCBI database, and only one (cytochrome P450) of the twenty most down-regulated genes had defined functions. Comparing the Dec. 15 and Jan. 15 libraries, three of the twenty most up-regulated genes (i.e., WRKY transcription factor 12, transcriptional factor TINY, blue copper protein precursor) and one (non-specific lipid-transfer protein 8-like) of the twenty most down-regulated genes had defined functions. Between the Jan. 15 and Feb. 15 libraries, three of the twenty most up-regulated genes had defined functions, including genes encoding LRR receptor-like serine, phd finger protein, and arabinogalactan-protein; none of the most down-regulated genes had defined functions.

### Functional classification of DEGs during dormancy stages

**Figure 6:**
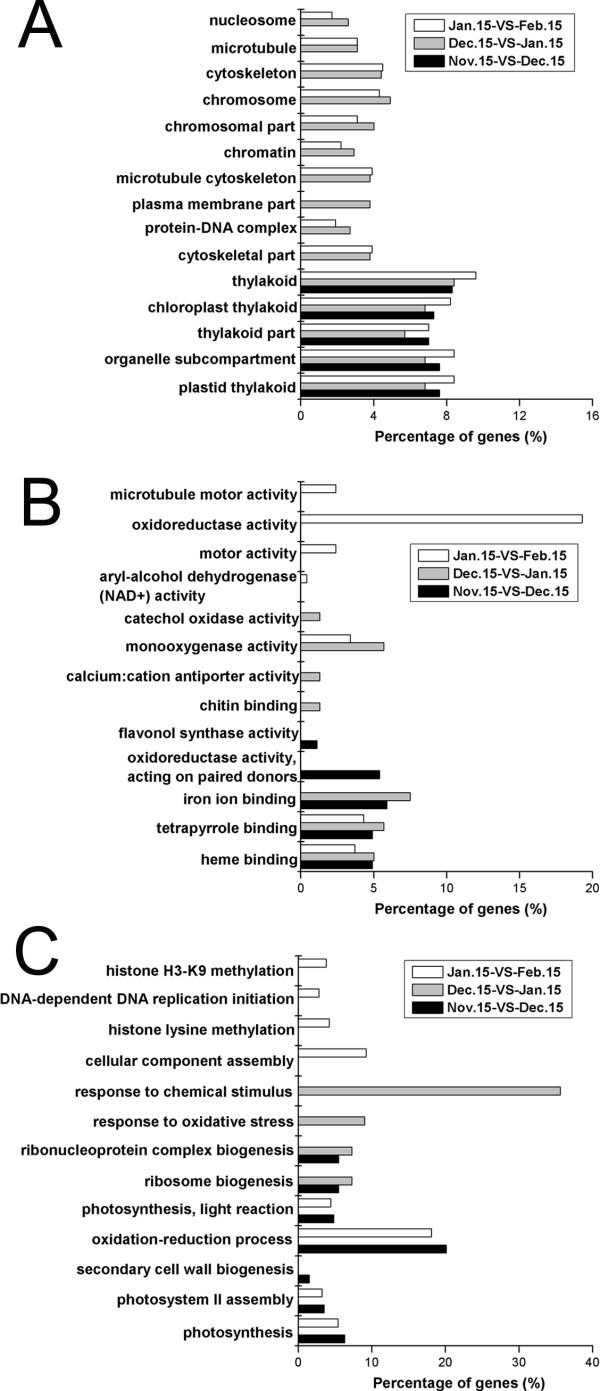
**Functional categories of differentially-expressed genes in the Gene Ontology.** GO categories that were significantly enriched (p-value < 0.05) were analyzed in pairwise comparisons (Nov. 15-VS-Dec. 15, Dec. 15-VS-Jan. 15, and Jan. 15-VS-Feb. 15). Percentages are based on the proportion of genes in each set. **A**: Cellular component; **B**: Molecular function; **C**: Biological process.

We used GO and KEGG assignments to classify the functions of DEGs in pairwise comparisons of cDNA libraries during pear dormancy, specifically the Nov. 15-VS-Dec. 15, Dec. 15-VS-Jan. 15, and Jan. 15-VS-Feb. 15 comparisons. The DEG functions were classed into three GO categories. In the cellular component category, ‘plastid thylakoid’, ‘organelle subcompartment’, ‘thylakoid part’, ‘chloroplast thylakoid’, and ‘thylakoid’ were significantly enriched (p-value <0.05) in all three pairwise comparisons. Ten other GO terms were significantly enriched only in the Dec. 15-VS-Jan. 15 and Jan. 15-VS-Feb. 15 comparisons (Figure 6A). In the molecular function category, only two GO, terms ‘heme binding’ and ‘tetrapyrrole binding’, were significantly enriched in all three pairwise comparisons, while ‘monooxygenase activity’ was significantly enriched in the Dec. 15-VS-Jan. 15 and Jan. 15-VS-Feb comparisons (Figure 6B). In the biological process category, no GO terms were significantly enriched in all three pairwise comparisons, but ‘photosynthesis’, ‘photosystem II assembly’, ‘oxidation-reduction process’, and ‘photosynthesis, light reaction’ were significantly enriched in the Nov. 15-VS-Dec. 15 and Jan. 15-VS-Feb. 15 comparisons, and ‘ribosome biogenesis’ and ‘ribonucleoprotein complex biogenesis’ were enriched in the Nov. 15-VS-Dec. 15 and Dec. 15-VS-Jan. 15 comparisons (Figure 6C).

In the Nov. 15-VS-Dec. 15, Dec. 15-VS-Jan. 15, and Jan. 15-VS-Feb. 15 comparisons, 666, 393, and 1,223 DEGs mapped to 104, 92, and 117 KEGG pathways, respectively. Of the 666 DEGs in the Nov. 15-VS-Dec. 15 comparison, 599 (89.9%) mapped to 15 pathways (Additional file 14). Remarkably, specific enrichment of unigenes was observed for 15 pathways involved in metabolic processes, such as phenylpropanoid biosynthesis, ether lipid metabolism, ribosome, cutin, suberine and wax biosynthesis, stilbenoid, diarylheptanoid and gingerol biosynthesis, endocytosis, glycerophospholipid metabolism, oxidative phosphorylation, zeatin biosynthesis, flavonoid biosynthesis, starch and sucrose metabolism, fructose and mannose metabolism, other glycan degradation, and pentose and glucuronate interconversions. Comparing the Dec. 15 and Jan. 15 libraries, 343 (87.3%) DEGs were identified in 6 pathways: ribosome, ether lipid metabolism, glycerophospholipid metabolism, endocytosis, plant-pathogen interaction, and metabolic pathways (Additional file 14). In the Jan. 15-VS-Feb. 15 comparison, 793 (64.8%) DEGs were significantly enriched in 15 pathways (Additional file 14).

### Clustering analysis of DEGs during dormancy stages

**Figure 7:**
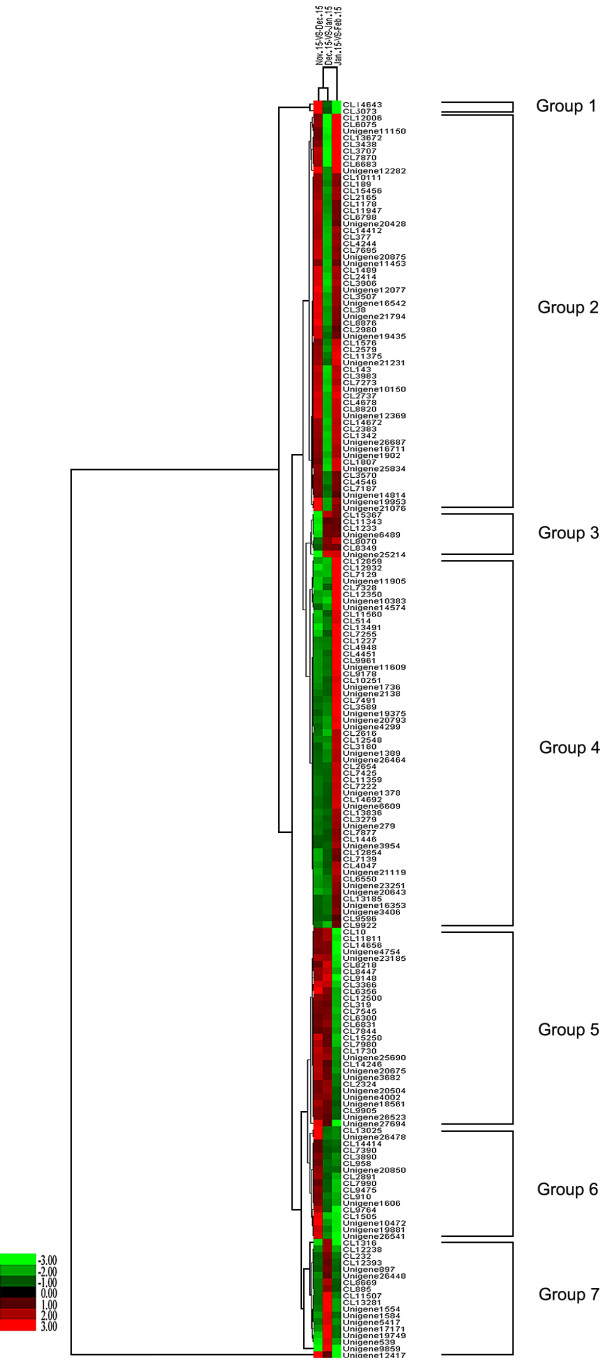
**Hierarchical clustering analysis of differentially-expressed genes during pear dormancy.** The log_2_Ratio for significantly differentially-expressed genes were used. Each column represents a comparison of samples between dates (e.g., Nov. 15-VS-Dec. 15), and each row represents a gene. Expression differences are shown in different colors; red indicates up-regulation and green indicates down-regulation. The 190 genes were classified into seven regulation patterns.

Genes with similar expression patterns are usually functionally correlated. To understand the expression patterns of 190 genes that were significantly-differentially expressed (Additional file 15) at different times in pear dormancy, cluster analyses of gene expression patterns in the Nov. 15-VS-Dec. 15, Dec. 15-VS-Jan. 15, and Jan. 15-VS-Feb. 15 comparisons were performed. These genes were arranged into seven groups (Figure 7). The largest group (Group 2) comprised the 60 (31.6%) genes, from CL 12006 to unigene 21076, that were up-regulated from Nov. 15 to Dec. 15, down-regulated between Dec. 15 and Jan. 15, then up-regulated again between Jan. 15 and Feb. 15. This group mainly included genes encoding proteins associated with ribosomes, such as 60S ribosomal protein, 40S ribosomal protein, translation initiation factor eIF-5A, ATP binding protein, and transaldolase.

The second largest group (Group 4) contained 56 (29.5%) genes, from CL 12859 to CL 9922, that were down-regulated from Nov. 15 to Jan. 15 then up-regulated between Jan. 15 and Feb. 15. This group mainly included genes encoding proteins associated with photosynthesis metabolism, such as chlorophyll A/B binding protein, photosystem I reaction center subunit III, photosystem I reaction center subunit IV A, plastocyanin A, chloroplast oxygen-evolving enhancer protein 1, ribulose bisphosphate carboxylase, cytochrome P450, and magnesium-protoporphyrin IX monomethyl ester cyclase. Additionally, zinc finger protein, SPL domain class transcription factor, and basic helix-loop-helix domain-containing protein were encoded.

The third largest group (Group 5) contained 30 (15.8%) genes, from CL 10 to unigene 27694, that were up-regulated from Nov. 15 to Jan. 15, then down-regulated between Jan. 15 and Feb. 15. This group mainly included genes encoding proteins associated with oxidation-reduction reaction and resistance, such as blue copper protein, 2-oxoglutarate-dependent dioxygenase, cytochrome P450, heat shock protein, glycine rich protein, and dehydrin 1.

In Group 6, 17 genes (8.9%), from CL 13025 to unigene 26541, were up-regulated between Nov. 15 and Dec. 15, but were down-regulated between Dec. 15 and Jan. 15 and between Jan. 15 and Feb. 15. This group mainly involved in genes encoding dehydration-responsive element-binding protein, galactinol synthase 1, and GA_2_-oxidase.

In Group 7, 18 genes (9.5%), from CL 1316 to unigene 12417, were down-regulated between Nov. 15 and Dec. 15 and between Jan. 15 and Feb. 15 but were up-regulated between Dec. 15 and Jan. 15. These genes encoded proteins including fructose-bisphosphate aldolase cytoplasmic isozyme, tyrosine-protein phosphatase SIW14, glycerol-3-phosphate transporter, NAC domain class transcription factor, WRKY domain class transcription factor, and AP2 domain class transcription factor.

In Group 3, seven genes were down-regulated from Nov. 15 to Dec. 15, but were up-regulated between Dec. 15 and Feb. 15. Finally, two genes (Group 1) were up-regulated from Nov. 15 to Dec. 15, but were down-regulated between Jan. 15 and Feb. 15. Of these nine genes, only 2-aminoethanethiol dioxygenase isoform 2 and transferase were definitely annotated.

### Gene expression analysis and Q-PCR validation

**Figure 8:**
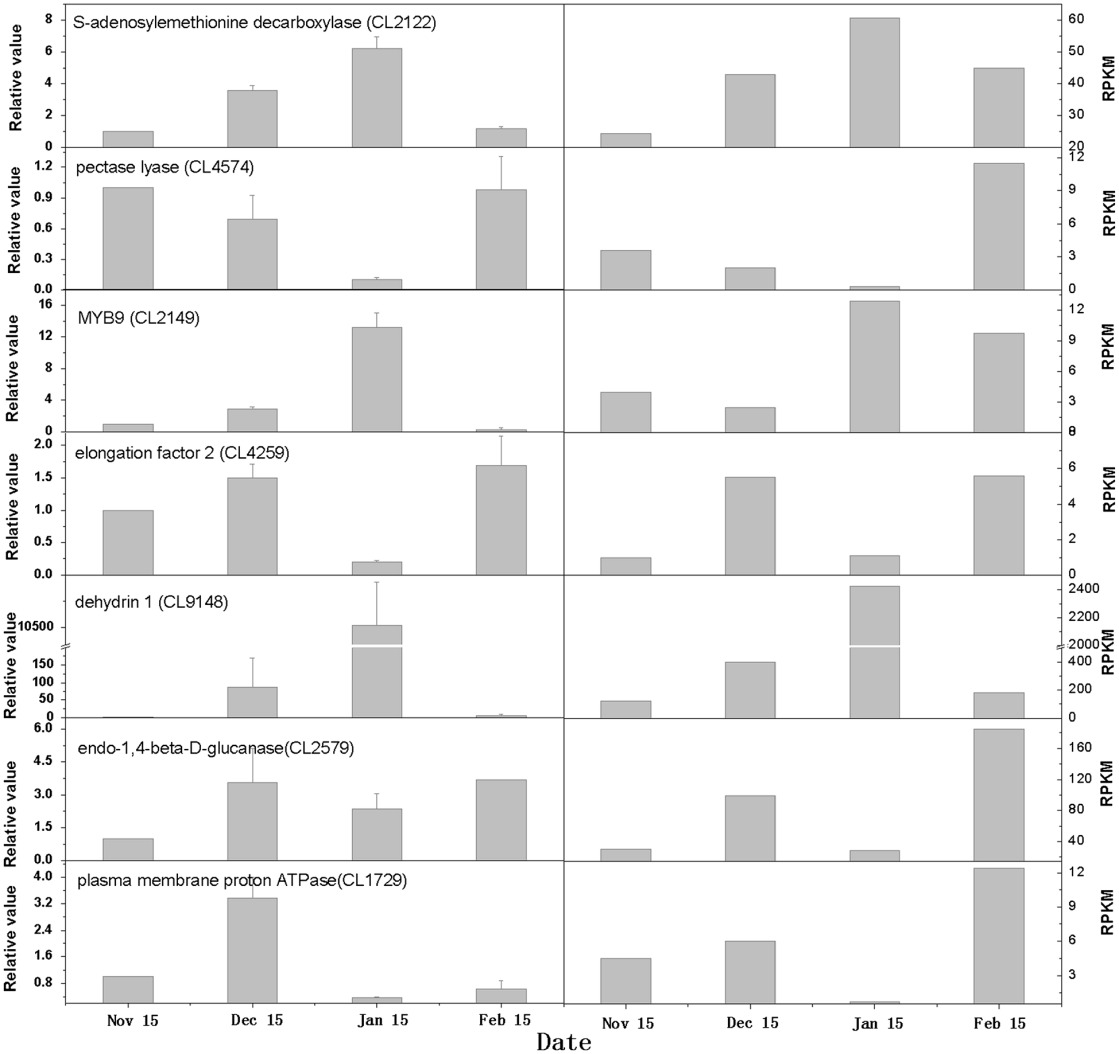
**Q-PCR validation of differential gene expression during dormancy.** The left y-axis indicates relative gene expression levels determined by Q-PCR. Bars represent the standard error (n = 3). The right y-axis indicates gene expression levels calculated by the RPKM method.

**Figure 9:**
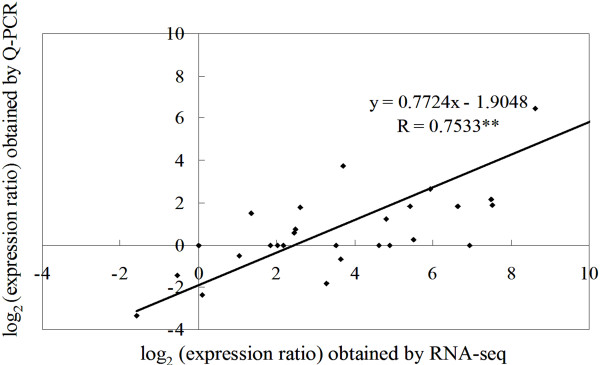
**Coefficient analysis between gene expression ratios obtained from RNA-seq and Q-PCR data.** The Q-PCR log_2_ values (expression ratios; y-axis) were plotted against dormancy stages (x-axis)**.** ** indicates a significant difference at p ≤ 0.01.

The RNA sampled at four times during bud dormancy provided templates for real-time quantitative PCR (Q-PCR) validation. We randomly selected seven DEGs to demonstrate the RNA-seq results (Figure 8). The Q-PCR data for these genes were basically consistent with the RNA-seq results of the four samples. Linear regression [(Q-PCR value) = α (RNA-seq value) + b] analysis showed a highly significant correlation (R = 0.7533^**^) which indicated good reproducibility between transcript abundance assayed by RNA-seq and the expression profile revealed by Q-PCR data (Figure 9).

### Phylogenetic analysis of dormancy-associated MADS-box (*DAM*) genes and their expression variations

**Figure 10:**
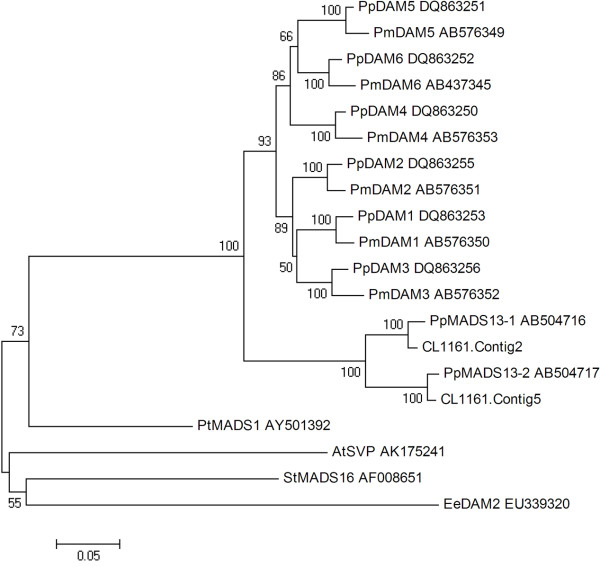
**Phylogenetic tree of two pear unigenes and 18 other MADS-box genes.** Two unigenes from ‘Suli’ pear (*Pyrus pyrifolia* white pear group; CL 1161.contig2 and CL 1161.contig5) were aligned with MADS-box genes from *Prunus persica* (DQ863253, DQ863255, DQ863256, DQ863250, DQ863251, and DQ863252), *Prunus mume* (AB576350, AB576351, AB576352, AB576353, AB576349, and AB437345), *Pyrus pyrifolia* (AB504716 and AB504717), *Populus tomentosa* (AY501392), *Arabidopsis thaliana* (AK175241), *Solanum tuberosum* (AF008651), and *Euphorbia esula* (EU339320). The tree was generated with MEGA 4.0.1 software using the neighbor–joining method. The numbers at each interior branch indicate bootstrap percentages from 1000 replicates.

A phylogenetic tree constructed using the nucleotide sequences of two unigenes and 18 other MADS-box genes (Figure 10) revealed that CL 1161.contig2 was more closely related to *PpMADS13-1* of *Pyrus pyrifolia*, whereas CL 1161.contig5 was more similar to *PpMADS13-2* of *P. pyrifolia*. Additionally, the results revealed that the pear *DAM* genes were more closely related to those of *Prunus* species (*Prunus persica* and *P. mume*). However, the pear *DAM* genes formed an independent subclade.

**Figure 11:**
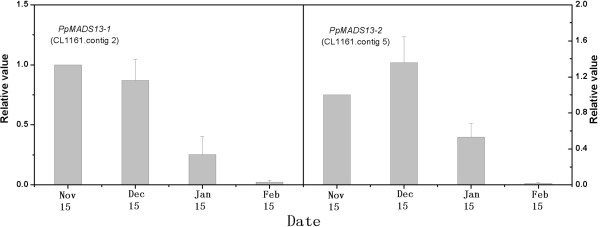
**Relative expression levels determined by Q-PCR of CL 1161.contig2 and CL 1161.contig5 during dormancy.** The left y-axis indicates the relative expression levels of CL 1161.contig2. The right y-axis indicates the relative expression levels of CL 1161.contig5. Bars represent the standard error (n = 3).

Based on the phylogenetic analysis, we selected two unigenes (CL 1161.contig2 and CL 1161.contig5) to analyze their expression variations during dormancy. The expression levels of both genes decreased with endodormancy release in lateral flower buds (Figure 11).

## Discussion

In this study, using RNA-Seq technology, a ‘Suli’ pear cDNA library and four DGE libraries (from samples collected on Nov. 15, Dec. 15, Jan. 15, and Feb. 15, 2010–2011) were constructed and used to screen DEGs during dormancy. Surprisingly, we obtained 69,393 unique sequences, and of which 51,448 could be annotated, in total 48,725 genes, including 14,531 clusters and 34,194 singletons. Until 23 May, 2012, there were only 4,339 expressed sequence tags (ESTs) and 2,837 nucleotide sequences of *Pyrus* plants deposited in GenBank. Several recent studies have used traditional EST analyses to study dormancy in other species. Horvath et al. (2008) and Mazzitelli et al. (2007) identified nearly 1,000 genes and 327 ESTs associated with bud dormancy in leafy spurge and raspberry, respectively, using cDNA microarrays [5,22]. Leida et al. (2010) identified nearly 400 ESTs associated with bud dormancy in peach by constructing four subtracted-cDNA libraries [46]. These ESTs participated in different metabolic pathways related to photoperiod, temperature, circadian clocks, water, energy, reactive oxygen species, and hormones [5,22,46]. To our knowledge, this is the first report to use RNA-Seq technique to identify large numbers of genes involved in different metabolic pathways in pear bud dormancy. Compared with traditional EST analyses, RNA-Seq was less expensive, more efficient, and allowed faster gene discovery in bud dormancy studies.

Through RNA-seq analysis, we found that the numbers and expression profiles of DEGs differed at different times during dormancy. A total of 1,978, 1,024, and 3,468 genes were differentially expressed between Nov. 15 and Dec. 15, Dec. 15 and Jan. 15, and Jan. 15 and Feb. 15, respectively. These results showed that the number of DEGs was fewer in the endodormant stage (Nov. 15 and Dec. 15) than in the ecodormant stage (Jan. 15 and Feb. 15), increased with endodormancy-release to reach a maximum by Feb. 15. Hedley et al. (2010) reported that gene activity was lowest in the early stages of dormancy and peaked around the time of bud break in blackcurrant (*Ribes nigrum* L.) [25]. By analyzing KEGG pathways, we found DEGs that participated in several different pathways. Some pathways (such as starch and sucrose metabolism, circadian rhythm, and flavonoid biosynthesis) had been previously correlated to bud break in other species [5,17,22,46], and some like phenylpropanoid biosynthesis, stilbenoid, diarylheptanoid and gingerol biosynthesis, zeatin biosynthesis, ether lipid metabolism, endocytosis, and glycerophospholipid metabolism were associated with bud break for the first time in this study. These data may suggest new research directions for understanding bud dormancy.

Some of the genes found in this work had been previously identified in other perennial plants. The *DAM* genes, widely described in perennial species such as leafy spurge [5], peach [9-12], raspberry [22], Japanese apricot [15], kiwifruit [24], and Japanese pear [31], are candidates for internal factors controlling endodormancy. In this study, we also found two *DAM* genes, and phylogenetic analysis revealed that CL 1161.contig2 was more closely related to *PpMADS13-1* of *Pyrus pyrifolia*, whereas CL 1161.contig5 was more similar to *PpMADS13-2* of *Pyrus pyrifolia*.Changes in the expression of CL 1161.contig2 and CL 1161.contig5 decreased with endodormancy release in lateral flower buds were consistent with the findings of earlier work comparing *PpMADS13-1* and *PpMADS13-2* gene expression in lateral leaf buds of Japanese pear [31], *PpDAM5* and *PpDAM6* in lateral vegetative buds and lateral flower buds of peach [10,11], and all *PmDAMs* (*PmDAM1PmDAM6*) in lateral vegetative buds of Japanese apricot [15]. Our study, along with previous studies, suggested that *DAM* genes might play significant roles in the regulation of bud dormancy in ‘Suli’ pear.

The accumulation of dehydrin (DHN) is known to be associated with freezing tolerance in plants [47]. Recent studies have reported that the accumulation of DHNs in woody plants correlates with seasonal transitions in dormancy and cold acclimation stages during winter [16,48], but characterizations of DHN genes expressed during dormancy are limited. Yakovlev et al. (2008) found a reduction in the transcript levels of most of the 15 DHNs that they cloned as Norway spruce neared bud-burst [48]. Garcia-Bañuelos et al. (2009) reported that transcripts of apple DHN were highly expressed in bark and bud tissues when the plant was dormant and cold hardy in midwinter, but were not expressed in early spring when cold hardiness was lost and buds were growing [49]. Recently, several studies have identified DHN genes that were activated by ABA and C- repeat binding factor (CBF) in response to abiotic stresses [14,50-52]. Intriguingly, in leafy spurge, ABA levels were elevated during endodormancy but dropped following the transition to ecodormancy [5]. Horvath et al. (2008) found that CBF genes involved in cold regulated were up-regulated during the transition from para- to endo-dormancy [5]. In the present study, one gene (CL 9148) encoding dehydrin showed significantly higher expression during the transition from endo- to eco-dormancy; thereafter, the expression level of this gene rapidly decreased, as indicated by DGE analysis and Q-PCR data. Based on previous studies, we speculated that DHN genes may act as signals and offer some protection for ‘Suli’ pear after the end of endodormancy, when pear often encounters unfavorable environmental conditions, such as cold. Therefore, more attention should be paid to ABA and CBF, which activate DHN genes, in future studies of transcriptional regulation related to the pear dormancy process.

Generally, sugar transport is thought to occur via H^+^/sugar symports that depend on a pH gradient generated by a plasma membrane H^+^-ATPase [53]. Gevaudant et al. (2001) examined expression of the four H^+^-ATPase genes and reported that the levels of three H^+^-ATPase gene mRNAs increased, whereas the level of one H^+^-ATPase gene decreased in vegetative buds of peach trees after dormancy release [54]. Mazzitelli et al. (2007) demonstrated that the plasma membrane H^+^-ATPase gene was highly expressed during the dormancy transition [22]. Our results showed that the expression of a plasma membrane H^+^-ATPase gene (CL 1729) was up-regulated in pear buds during the endodormant maintenance period, down-regulated during endodormancy-release, and then up-regulated again. The expression patterns of plasma membrane H^+^-ATPase in ‘Suli’ pear were different from those of peach and raspberry buds, perhaps due to species-level or tissue-level differences.

In the present study, some genes encoding galactinol synthase (CL 9475), plastocyanin A (CL 1227), chlorophyll A/B binding protein (CL 7129, CL 514, CL 4948, CL 9178, CL 9961, CL 3279), and S-adenosylemethionine decarboxylase (CL 2122) were differentially expressed. Of these, chlorophyll A/B binding protein and S-adenosylemethionine decarboxylase were previously reported in other perennial plants [46,55]. The expression levels of these genes changed significantly during the dormancy process. Thus, these genes may play roles in the regulation of bud dormancy in ‘Suli’ pear.

In addition, differentially-regulated transcription factors were identified in this study, including AP2 (unigene 1554), Zn-finger (unigene 16353), NAC (CL 7187), WRKY (unigene 19749), SPL (CL 3589), and bHLH (CL 12548). Of these, AP2, Zn-finger, and NAC were previously reported in leafy spurge [5] and peach [46]. Based on DGE analysis, the expression levels of the genes encoding these transcription factors significantly changed during dormancy in ‘Suli’ pear.

Although the molecular mechanisms associated with dormancy transitions in pear trees remain largely unknown, the present transcriptome analysis provided valuable information that could facilitate future studies on the detailed molecular functions of genes related to pear bud dormancy.

## Conclusions

We obtained transcriptome data that provided comprehensive sequencing and DGE profiling data for a dynamic view of transcriptomic variation during the dormancy stage in pear. Physiological processes such as phenylpropanoid biosynthesis, stilbenoid, diarylheptanoid and gingerol biosynthesis, zeatin biosynthesis, ether lipid metabolism, endocytosis, glycerophospholipid metabolism, photosynthesis, phenylalanine metabolism, starch and sucrose metabolism were all differentially regulated during bud dormancy. Approximately 190 genes involved in many metabolic processes were significant differentially regulated during bud dormancy. Genes related to bud dormancy and their expression profiles at four time-points during dormancy were analyzed further. This offered new insights into the molecular mechanisms underlying pear bud dormancy. This provided a relatively complete molecular platform for future research on the progression of pear bud dormancy. To our knowledge, this work is the first to study pear bud dormancy using RNA-Seq.

## Methods

### Plant materials

‘Suli’ pear cultivars grafted onto *Pyrus betulaefolia* Bunge rootstocks were obtained from the Dangshan Germplasm Resources Center (Dangshan County, Anhui Province, China). Pear trees were 10 years old and were considered to be in the adult phase. Trees used in the experiment were not pruned or chemically treated during the experimental period. All samples were collected from the same trees at each stage. The current season’s growth shoots were collected on Nov. 15, Dec. 15, Jan. 15, Jan. 25and Feb. 15 from Nov. 2010 through Feb. 2011. Lateral flower bud samples were stored immediately in liquid N_2_ and then at −80°C until RNA extraction after picking.

### Dormancy status of lateral flower buds

The dormancy status of lateral flower buds at four collection dates (Nov. 15, Dec. 15, Jan. 15, and Feb. 15) was estimated by evaluating excised shoots from field-grown trees. To measure the percentage of bud break, 12 current season’s growth shoots, with lengths of about 60 cm and bearing apical buds and 10–12 lateral flower buds, were collected and placed in water in 500 mL vials in a phytotron kept at day/night 25 ± 1/18 ± 1°C, with a 12-h photoperiod of white light (320 μ photon mol m^-2^ s^-1^) and 75% humidity. The water in the vials was changed and the basal ends of the shoots were cut every 2–3 d. After 21 d, the dormancy status was valued by percentage bud break; the beginning of bud break was defined as green leaf tips enclosing visible flowers. Lateral flower buds of shoots with bud break percentages < 50% were considered to remain in the endodormant stage.

### RNA extraction, library preparation and RNA-seq

Thirty shoots (three biological replicates, with 10 shoots per replicate) with lengths of about 60 cm and bearing 10–12 lateral flower buds were sampled at each stage (Nov. 15, Dec. 15, Jan. 15, Jan. 25, and Feb. 15). The buds were sampled from three biological replications of each stage and produced an independent pool. Total RNA was extracted from lateral flower buds using the pine tree extraction protocol [56]. The transcriptome assembly library was pooled by mixing equal quantities of RNA of five dormancy stages. The four DGE libraries consisted of separate RNA extracts from buds of four different dormancy stages, i.e., Nov. 15, Dec. 15, Jan. 15, and Feb. 15. Each library was pooled by mixing equal quantities of RNA from three biological replications for each stage. Each pool was sequenced once technically since the RNA-seq data are highly replicable with relatively little technical variation [57]. The following protocols were performed by staff at the Beijing Genome Institute (BGI; Shenzhen, China). RNA integrity was confirmed with a 2100 Bioanalyzer (Agilent Technologies, Santa Clara, CA, USA). Oligo-(dT) magnetic beads were used to isolate poly-(A) mRNA from total RNA, and mRNA was fragmented in fragmentation buffer. Using these short fragments (≈200 bp) as templates, random hexamer-primers were used to synthesize first-strand cDNA. Second-strand cDNA was synthesized using buffer, dNTPs, RNaseH, and DNA polymerase I. Short double-stranded cDNA fragments were purified with QiaQuick PCR extraction kit (Qiagen, Venlo, The Netherlands), resolved with EB buffer for end reparation and adding poly (A), then ligated to sequencing adapters. After purification via agarose gel electrophoresis, suitable fragments were enriched by PCR amplification before library sequencing using Illumina HiSeq™ 2000 (San Diego, CA, USA).

### *De novo* assembly and function annotation

Raw sequence data in fastq format were filtered to remove reads containing adaptors, reads with more than 5% unknown nucleotides, and low-quality reads with more than 20% bases of quality value ≤ 10. Only clean reads were used in the following analysis. The sequences from the Illumina sequencing were deposited in the NCBI Sequence Read Archive (Accession SRX147917). First, transcriptome *de novo* assembly was carried out by BGI using the short-read assembly program Trinity [58] with the following parameters: min_contig_length = 100, min_glue = 2, group_pairs_distance = 250, path_reinforcement_distance = 75, bfly_opts = '-V 5 --edge-thr = 0.05 --stderr', min_kmer_cov = 2. Meanwhile, all reads of approximately 100 M (i.e. transcriptome sequencing reads and RNA-Seq reads) were mapped back to the apple genome reference (ftp://ftp.jgi-psf.org/pub/JGI_data/phytozome/v8.0/Mdomestica/assembly/Mdomestica_196.fa.gz) to identify continuous gene regions using SOAPsplice software (Release 1.9; http://soap.genomics.org.cn/soapsplice.html). Secondly, we realigned all the transcripts onto the reference genome. When more than one transcript were placed in one gene region and they each other had an overlap less than 24 bp, we connected them into a longer transcript. A total of 31,727 transcripts assembled by Trinity were connected into 19,309 transcripts. Then the redundancy of unigenes was removed by TGICL (v.2.1) with options ‘-l 40 -v 25’. Finally based on gene family clustering, the unigenes were divided into two classes: clusters and singletons. The former was prefixed with 'CL' and the latter with 'unigene'. The id number of each unigene followed this prefix. In a cluster, the similarity between unigenes was more than 70%.

Blastx alignment (E-value < 10^-5^) between unigenes and protein databases such as nr, Swiss-Prot, KEGG, and GO was performed, and the best-aligning results were used to determine the sequence direction of unigenes. When different databases conflicted, the results were prioritized in the order: nr, Swiss-Prot, KEGG, and GO. When a unigene did not align to any of the databases, ESTScan [59] was used to decide its sequence direction. GO annotation was analyzed by Blast2GO software (v.2.5.0). KEGG pathway annotation was performed using Blastall software against the KEGG database. The assembled sequences could be searched using the Gene-ID listed in Additional materials (Additional file 3).

### Analysis and mapping of DGE reads

Raw sequence data in fastq format were filtered to remove reads containing adaptors, reads with more than 10% unknown nucleotides, and low-quality reads with more than 50% bases of quality value ≤ 5. The sequences from the DGE analysis were deposited in the NCBI Sequence Read Archive under accession numbers SRX148326 (Nov. 15), SRX148327 (Dec. 15), SRX148328 (Jan. 15), and SRX148329 (Feb. 15). Clean reads were mapped to our transcriptome reference database using the short oligonucleotide analysis package SOAPaligner/soap2 [60], allowing mismatches of no more than two bases. The unique mapped reads were used in subsequent analyses. For gene expression analysis, the number of unique-match reads was calculated and then normalized to RPKM (reads per kb per million reads) [61]. The RPKM method eliminates the influence of different gene lengths and sequencing discrepancies on the calculation of gene expression, so that the calculated gene expressions can be directly compared among samples.

### Evaluation of DGE libraries

A statistical analysis of the frequency of each unique-match read in each DGE library was performed to compare gene expression at different times in pear dormancy using the method described by Audic et al. [62]. The *P* value was used to identify genes expressed differentially between each samples following the formula below, in which N1 and N2 represent the total numbers of unique-match reads in Samples 1 and 2, respectively, and gene A contained x and y unique-match reads mapped to Samples 1 and 2, respectively. Enriched *P*-values were calculated according to the hypergeometric test [62]:

(1) $$2\sum_{i=0}^{i=y}p\left(i|x\right)\left(\mathrm{if}\sum_{i-0}^{i=y}p\left(i|x\right)\leq 0.5\right)\;\mathrm{or}\;2\times \left(1-\sum_{i=0}^{i=y}p\left(i|x\right)\right)\left(\mathrm{if}\sum_{i=0}^{i=y}p\left(i|x\right)>0.5\right)$$

(2) $$p\left(y|x\right)={\left(\frac{N_{2}}{N_{1}}\right)}^{y}\frac{\left(x+y\right)!}{x!y!{\left(1+\frac{N_{2}}{N_{1}}\right)}^{\left(x+y+1\right)}}$$

The false discovery rate (FDR) was used to determine the *P*-value threshold in multiple testing [63]. Briefly, assuming that R differentially expressed genes had been selected, S genes of those were really shown differential expression, whereas the other V genes actually indicated no difference which were false positive. If the error ratio (Q = V/R) was required to remain below a specified cutoff (0.01), the FDR value should not exceed 0.01. FDR-values were calculated according to the Benjamini and Hochberg algorithm [63]:

(3) $$\mathrm{FDR}=E\left(Q\right)=E\left\{V/\left(V+S\right)\right\}=E\left(V/R\right)$$

We used FDR ≤ 0.001, the absolute value of log_2_Ratio ≥ 1, and the RPKM value of each gene for either sample over 10 as the thresholds to judge the significance of gene expression differences, where log_2_Ratio indicates the degree of differential expression between two samples and was the ratio of RPKM values for the treatment and control samples. This analysis found genes with significantly differential expression among samples prior to GO function and KEGG pathway analyses.

### Clustering of gene expression profiles

Hierarchical cluster analysis of 190 gene expression patterns was performed with cluster [64] and Java Treeview [65] software. The log_2_Ratio for each gene was used for the hierarchical clustering analysis.

### Phylogenetic analysis

A phylogenetic tree was constructed based on the nucleotide sequences of two unigenes and 18 additional *DAM* genes. The tree was generated with MEGA (v. 4.0.1) [66] software, using the neighbor-joining method.

### Real-time quantitative RT-PCR analysis

Total RNA used for Q-PCR analysis was extracted from lateral flower buds of four different dormancy stages, i.e., Nov. 15, Dec. 15, Jan. 15, and Feb. 15, using three biological replicates of about 300 buds. Total RNA was extracted as described above, genomic DNA was removed with DNase I, and total RNA concentration was measured. First-strand cDNA was synthesized from 4 μg of DNA-free RNA using the Revert Aid™ First Strand cDNA Synthesis Kit (Fermentas, Glen Burnie, MD, USA) according to the manufacturer’s instructions. The cDNA was used as the template for Q-PCR. Primer sequences (designed using primer 3, http://frodo.wi.mit.edu/cgi-bin/primer3/primer3_www.cgi) are listed in Additional file 16. The Q-PCR mixture (20 μl total volume) contained 10 μl of SYBR Premix ExTaq™ (Takara, Kyoto, Japan), 0.4 μl of each primer (10 μM), 2 μl of cDNA, and 7.2 μl of RNase-free water. The reactions were performed on a LightCycler 1.5 instrument (Roche, Basel, Switzerland) according to the manufacturer’s instructions. The two-step Q-PCR program began with 30 seconds at 95°C, followed by 40 cycles of 95°C for 5 seconds and 60°C for 20 seconds. Template-less controls for each primer pair were included in each run. The specificity of Q-PCR primers was confirmed by melting curve and sequencing of Q-PCR products. Expression was calculated as 2^-∆ ∆ Ct^ and normalized to that of the actin gene (*PpActin*, JN684184) [67], and data were managed with the Data Processing System (DPS, v. 7.05, Zhejiang University, Hangzhou, China).

## Abbreviations

BGI: Beijing Genome Institute; BLAST: Basic local alignment search tool; DAM: Dormancy-associated MADS-box; DEG: Differentially-expressed gene; DGE: Digital gene expression; DHN: Dehydrin; DPS: Data processing system; EST: Expressed sequence tag; FDR: False discovery rate; KEGG: The Kyoto encyclopedia of genes and genomes database; KO: KEGG Orthology ids; MEGA: Molecular evolutionary genetics analysis; nr: NCBI non-redundant database; Q-PCR: Real-time quantitative PCR; RNA-Seq: RNA-sequencing; RPKM: Reads per kb per million reads; SOAP: Short oligonucleotide analysis package.

## Competing interests

The authors declare that they have no competing interests.

## Authors' contributions

GQL carried out the experiment, analyzed the data and drafted the manuscript. WSL carried out bioinformatics analysis. PHZ carried out the experiment and participated in data analysis. TX participated in data analysis. LJC participated in data analysis. DFL carried out the experiment. HS helped to draft the manuscript. YWT initiated and supervised the study. All authors read and approved the final manuscript.

## Supplementary Material

Additional file 1 **Overview of 'Suli' pear (*Pyrus pyrifolia*white pear group) transcriptome sequencing and assembly.** (**A**) Size distribution of Illumina sequencing contigs. (**B**) Size distribution of unigenes after paired-end and gap filling. Click here for file

Additional file 2 Primers used in RT-PCR to evaluate the quality of unigene sequencing data. Click here for file

Additional file 3 Top BLAST hits from the four public database (nr, SwissProt, KEGG, and GO). Click here for file

Additional file 4 The 29,500 unigenes assigned to 128 KEGG pathways. Click here for file

Additional file 5 **Sequencing saturation analysis of the four libraries.** All libraries approached saturation as the number of reads approached 5 million, as indicated by the decline in the number of new genes detected. Click here for file

Additional file 6 **Distribution of reads on reference genes.** All libraries showed good levels of randomness, with the number of reads evenly distributed throughout the transcriptomes. Click here for file

Additional file 7 **Differentially-expressed genes between the Nov. 15 and Dec. 15 libraries.** Genelength: length of all exon in gene. Expression: unique reads of aligned reads. RPKM: Reads Per Kb per Million reads. log_2_ Ratio: log_2_ (Dec. 15-RPKM/Nov. 15-RPKM). Up-Down-Regulation (Dec. 15/Nov. 15): Dec. 15 is a up/down regulation relative to Nov. 15. P-value: pvalue for hypothesis testing. FDR: false discovery rate. We used FDR ≤ 0.001 and the absolute value of log_2_ Ratio ≥ 1 as the threshold to judge the significance of gene expression difference. Click here for file

Additional file 8 **Differentially-expressed genes between the Nov. 15 and Jan. 15 libraries.** Genelength: length of all exon in gene. Expression: unique reads of aligned reads. RPKM: Reads Per Kb per Million reads. log_2_ Ratio: log_2_ (Jan. 15-RPKM/Nov. 15-RPKM). Up-Down-Regulation (Jan. 15/Nov. 15): Jan. 15 is a up/down regulation relative to Nov. 15. P-value: pvalue for hypothesis testing. FDR: false discovery rate. We used FDR ≤ 0.001 and the absolute value of log_2_ Ratio ≥ 1 as the threshold to judge the significance of gene expression difference. Click here for file

Additional file 9 **Differentially-expressed genes between the Nov. 15 and Feb. 15 libraries.** Genelength: length of all exon in gene. Expression: unique reads of aligned reads. RPKM: Reads Per Kb per Million reads. log_2_ Ratio: log_2_ (Feb. 15-RPKM/Nov. 15-RPKM). Up-Down-Regulation (Feb. 15/Nov. 15): Feb. 15 is a up/down regulation relative to Nov. 15. P-value: pvalue for hypothesis testing. FDR: false discovery rate. We used FDR ≤ 0.001 and the absolute value of log_2_ Ratio ≥ 1 as the threshold to judge the significance of gene expression difference. Click here for file

Additional file 10 **Differentially-expressed genes between the Dec. 15 and Jan. 15 libraries.** Genelength: length of all exon in gene. Expression: unique reads of aligned reads. RPKM: Reads Per Kb per Million reads. log_2_ Ratio: log_2_ (Jan. 15-RPKM/Dec. 15-RPKM). Up-Down-Regulation (Jan. 15/Dec. 15): Jan. 15 is a up/down regulation relative to Dec. 15. P-value: pvalue for hypothesis testing. FDR: false discovery rate. We used FDR ≤ 0.001 and the absolute value of log_2_ Ratio ≥ 1 as the threshold to judge the significance of gene expression difference. Click here for file

Additional file 11 **Differentially-expressed genes between the Dec. 15 and Feb. 15 libraries.** Genelength: length of all exon in gene. Expression: unique reads of aligned reads. RPKM: Reads Per Kb per Million reads. log_2_ Ratio: log_2_ (Feb. 15-RPKM/Dec. 15-RPKM). Up-Down-Regulation (Feb. 15/Dec. 15): Feb. 15 is a up/down regulation relative to Dec. 15. P-value: pvalue for hypothesis testing. FDR: false discovery rate. We used FDR ≤ 0.001 and the absolute value of log_2_ Ratio ≥ 1 as the threshold to judge the significance of gene expression difference. Click here for file

Additional file 12 **Differentially-expressed genes between the Jan. 15 and Feb. 15 libraries.** Genelength: length of all exon in gene. Expression: unique reads of aligned reads. RPKM: Reads Per Kb per Million reads. log_2_ Ratio: log_2_ (Feb. 15-RPKM/Jan. 15-RPKM). Up-Down-Regulation (Feb. 15/Jan. 15): Feb. 15 is a up/down regulation relative to Jan. 15. P-value: pvalue for hypothesis testing. FDR: false discovery rate. We used FDR ≤ 0.001 and the absolute value of log_2_ Ratio ≥ 1 as the threshold to judge the significance of gene expression difference. Click here for file

Additional file 13 **The top 20 most up-regulated and down-regulated DEGs between samples (Nov. 15-VS-Dec. 15, Dec. 15-VS-Jan. 15 and Jan. 15-VS-Feb. 15).** RPKM: Reads Per Kb per Million reads. DEGs: Differentially-expressed genes with the expression fold (log_2_Ratio ≥ 2) and false discovery rate (FDR ≤ 10^-5^) as the threshold. Click here for file

Additional file 14 **KEGG pathways significantly enriched during dormancy.** KEGG pathways significantly enriched were defined as pathways with Q-value ≤ 0.05. Click here for file

Additional file 15 **190 genes differentially expressed during dormancy.** In total, 190 genes were found that were significantly differentially-expressed genes between Nov. 15 and Dec. 15, between Dec. 15 and Jan. 15, and between Jan. 15 and Feb. 15. 190 genes with annotations and their expression fold between Nov. 15 and Dec. 15, between Dec. 15 and Jan. 15, and between Jan. 15 and Feb. 15. Click here for file

Additional file 16 Primers used in Q-PCR to validate differential expression during pear dormancy. Click here for file
