# Supplementary figures and images for: Transcriptomic analysis of ‘Suli’ pear (Pyrus pyrifolia white pear group) buds during the dormancy by RNA-Seq
Source: BMC Genomics. 2012 Dec 12;13:700. doi: 10.1186/1471-2164-13-700 (PMC3562153; doi:10.1186/1471-2164-13-700)

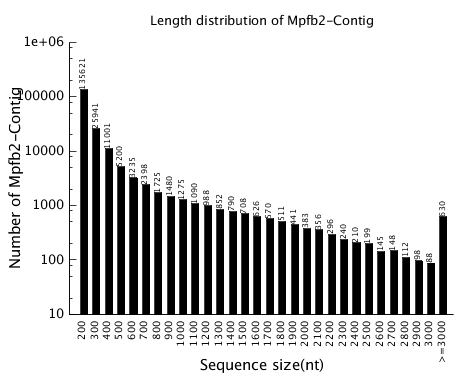


A


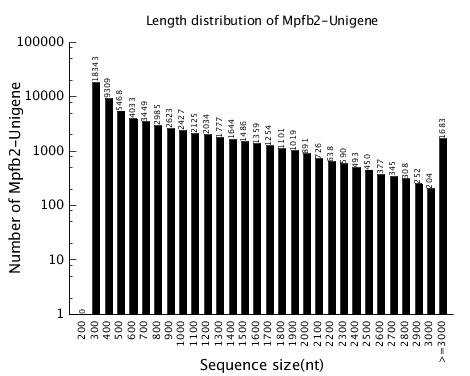


B

Supplement: Additional file 1 — Overview of 'Suli' pear (Pyrus pyrifoliawhite pear group) transcriptome sequencing and assembly. (A) Size distribution of Illumina sequencing contigs. (B) Size distribution of unigenes after paired-end and gap filling. [file 1471-2164-13-700-S1.doc]

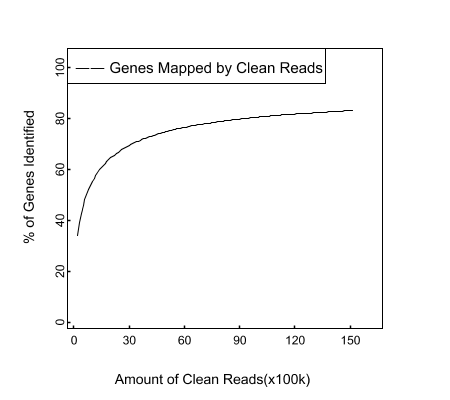

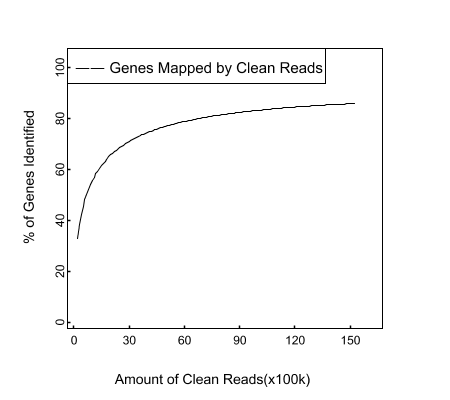


Nov.15 Dec.15


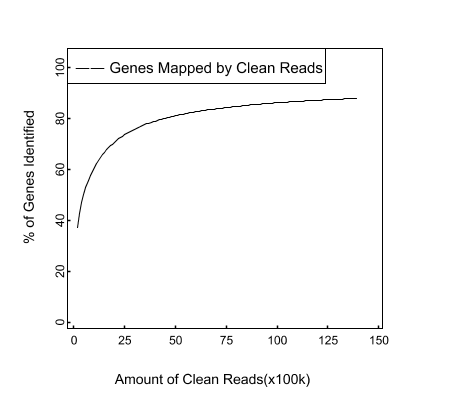


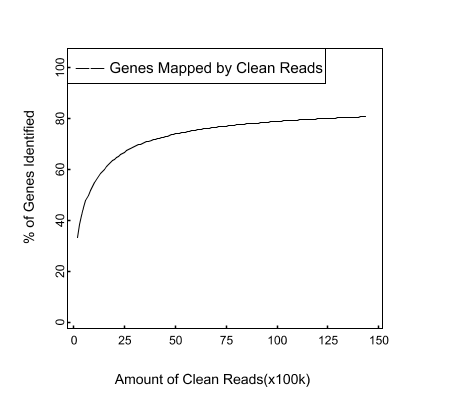


Jan.15 Feb.15

Supplement: Additional file 5 — Sequencing saturation analysis of the four libraries. All libraries approached saturation as the number of reads approached 5 million, as indicated by the decline in the number of new genes detected. [file 1471-2164-13-700-S5.doc]

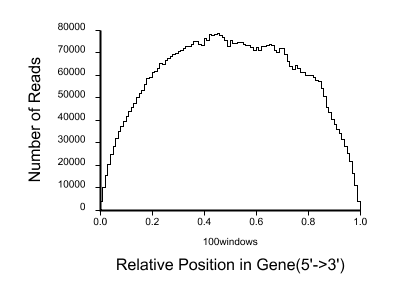

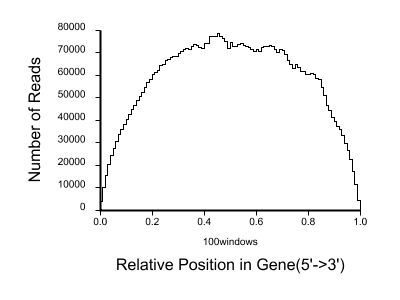


Nov.15 Dec.15


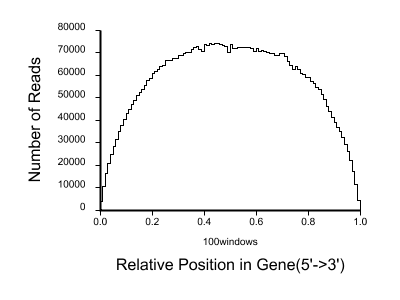


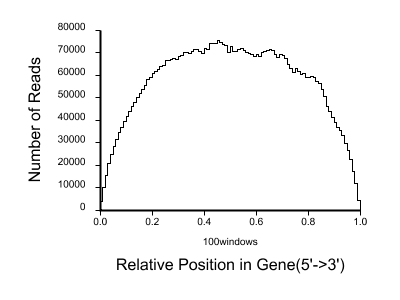


Jan.15 Feb.15

Supplement: Additional file 6 — Distribution of reads on reference genes. All libraries showed good levels of randomness, with the number of reads evenly distributed throughout the transcriptomes. [file 1471-2164-13-700-S6.doc]
