# Supplementary material for: Transcriptomic analysis of ‘Suli’ pear (Pyrus pyrifolia white pear group) buds during the dormancy by RNA-Seq
Source: BMC Genomics. 2012 Dec 12;13:700. doi: 10.1186/1471-2164-13-700 (PMC3562153; doi:10.1186/1471-2164-13-700)
Supplement: Additional file 2 — Primers used in RT-PCR to evaluate the quality of unigene sequencing data. [file 1471-2164-13-700-S2.doc]

**Primers used in RT-PCR for evaluating the quality of sequencing data of unigenes**

| GeneID | Forward primer (5′ to 3′) | Reverse primer (5′ to 3′) | Product size (bp) |
| --- | --- | --- | --- |
| CL10183.Contig1 | TATTTACGGAACTCAGGATC | CTCATTTACAGCCGAAGC | 581 |
| CL12069.Contig1 | GCTGTGCTCAAATCCGTA | AGAAGAAATCGCACAAGC | 871 |
| Unigene239 | GCAAGTCGGTCTCATCCT | CTCTGCGAAATACAACGTC | 457 |
| CL14990.Contig1 | AAACCACCACAACCACCG | CGTCCCACTTTCATTCCA | 393 |
| CL9131.Contig3 | AAATCATCCACCTCAACG | ATCGCAAAGAAGGAACAC | 1250 |
| CL7662.Contig1 | TGGTTCTCAGGGTGGTTC | TGACATCACTCGCTTTGC | 1217 |
| CL11513.Contig2  CL4650.Contig1 | CTTGGCTCGGGCTTGCTC  CACCTCCGTGAAGTATCCC | ATTTCCAGCGAGGGCAGA  CGAAAGGTCGATCCAGATA | 660  756 |
