# Supplementary material for: Transcriptomic analysis of ‘Suli’ pear (Pyrus pyrifolia white pear group) buds during the dormancy by RNA-Seq
Source: BMC Genomics. 2012 Dec 12;13:700. doi: 10.1186/1471-2164-13-700 (PMC3562153; doi:10.1186/1471-2164-13-700)
Supplement: Additional file 16 — Primers used in Q-PCR to validate differential expression during pear dormancy. [file 1471-2164-13-700-S16.doc]

Primer sequences used for Q-PCR

| GeneID | Forward primer (5′ to 3′) | Reverse primer (5′ to 3′) |
| --- | --- | --- |
| CL2122.contig1 | TAATGGTAACCGTAGAATG | TGAATACAACAGGCATAG |
| CL4574.contig1 | GGAGCAGGTACAGAAGCG | GAGGAGGAAGACGAAGATAGAG |
| CL2149.contig4 | GCTTGACTCTGAGGATTG | CACTTGGTTATTTATGTTAC |
| CL4259.contig1 | TTTCGCCCTTGATGTTAG | CATTATCCGTATCTTTCGTG |
| CL9148.contig1 | ACCGAACACTACGCAATA | CAAGCGAGCAAACTAAAT |
| CL2579.contig1 | AGCTCCATGAAACAAAGG | TAAACCCGTCTCATCACT |
| CL1729.contig2  CL1161.contig2  CL1161.contig5 | ACGGTGTCAACGATGCTC  CCTAAGACCCCTCAACGA  CGAAGGGGTAGCAAGAAA | CCTTCATACGCTGGAAAA  ACAGCAACTTCAGATTCACA  ACAGCAACTTCAGATTCACA |
